# Time trend analysis of social inequalities in psychological distress among young adults before and during the pandemic: evidence from the UK Household Longitudinal Study COVID-19 waves

**DOI:** 10.1136/jech-2021-217266

**Published:** 2021-10-29

**Authors:** Thierry Gagné, Alita Nandi, Ingrid Schoon

**Affiliations:** 1 Epidemiology and Public Health, UCL, London, UK; 2 Institute for Social and Economic Research, University of Essex, Colchester, UK; 3 Social Research Institute, UCL, London, UK

**Keywords:** social sciences, public health, mental health, longitudinal studies, health inequalities

## Abstract

**Background:**

Despite concerns about mental health problems among those aged 16–24 in England, which social groups have been most at risk, both over the past decade and during the COVID-19 pandemic, remains unclear.

**Methods:**

We examined trends in psychological distress among young adults 16–24 years old in England using data from the UK Household Longitudinal Study. Using longitudinal data as repeated cross-sectional waves, we examined differences over time in mean General Health Questionnaire (GHQ) scores from wave 1 (2009–2010) to wave 10 (2018–2019) and six COVID-19 waves collected between April and November 2020, by economic activity, cohabitation with parents, parental education, area deprivation, ethnicity, age and sex.

**Results:**

Compared with 2009–2010, increases in GHQ scores in 2018–2019 were higher in women than men (2.1 vs 1.3), those aged 16–18 than aged 22–24 (2.6 vs 0.9), those from white UK group versus other ethnic minorities, and those out of the labour force (3.6) or employed part time (2.2) than those employed full time (0.8). Compared with 2018–2019, psychological distress in 2020 also further increased among young adults residing in the most deprived areas (4.1 vs 1.2 in the least deprived areas). In 2020, losing one’s job or most of one’s work hours was associated with higher psychological distress and attenuated the differences between deprivation quartiles by 17%.

**Conclusion:**

In England, inequalities in psychological distress among young adults may have changed and increased during the COVID-19 pandemic. Investing in opportunities for young adults, particularly in more deprived areas, may be key to improve population levels of mental health.

## Introduction

In the wake of the COVID-19 pandemic, there has been a massive increase in psychological distress and mental health problems among young adults aged 16–24 in England, particularly in women.^1–3^ This exacerbated a crisis which already disproportionally affected this age group, with 1 in 10 men and 1 in 4 women aged 16–24 likely to be experiencing a mental health disorder before the pandemic.^4^ Mental health conditions emerging in this life period have a high risk of persisting if not treated and/or properly managed, and are predictive of a range of negative social and economic outcomes if they persist at later ages.^4 5^

Although mental health is strongly affected by social factors at the personal, family and community levels,^6^ there is little evidence on the distribution of mental health in those aged 16–24 compared with other age groups.^7–9^ Beyond what may be gleaned from studies in adult samples, there is also a paucity of evidence on inequalities in mental health changes during the pandemic in this age group, despite evidence that they have been among those most affected.^3 10 11^ The changes which have affected young adults over the past decade and during the pandemic are however likely to drive in inequitable ways the distribution of mental health in this age group.

Young adulthood is characterised by new, interlinked social role transitions, including establishing oneself in the labour market and living independently.^12 13^ In particular, employment offers young adults an important opportunity to fulfil their basic psychological needs and develop their agency and a positive social identity.^14^ Whereas employment in this age group has been defined by declining wages and work conditions over time, young adults not in employment, education or training continue to report the worst mental health outcomes.^15^ In response to these worsening conditions, many have delayed the move into independent living and family transitions over time.^13 16^ These conditions also led more to move back home, which has been associated with increased mental health problems, particularly when due to unemployment.^16–18^

Many sociodemographic factors shape these transitions and their relationship with mental health. Whereas participation in higher education increased across all social groups over time, in particular among women, young adults from less privileged families remain less likely to go to university, and those who do remain more likely to pursue lower-paying degrees and move into jobs for which they are overqualified.^19^ Independent of family background, growing up in a deprived area is also linked to early exits from education, longer unemployment spells and more mental health problems in young adulthood.^20–22^ Regarding ethnicity, whereas minority youths have had similar or better educational outcomes compared with white British youths in more recent years, inequalities in work conditions and earnings persist.^23^ Evidence on ethnic differences in mental health among young adults, however, is lacking in the UK. In adolescents, studies found better mental health among minority groups compared with white British people, supporting a potential ‘race paradox’ (ie, that ethnic minorities report better health) for mental distress in this age group.^24^

Evidence from the start of the pandemic has highlighted young adults to be at high risk of job loss.^25^ Partially supporting its impact on mental health, young adults who felt worse off financially compared with before the outbreak also reported more stress in May 2020.^26^ Many who kept their job also faced challenges, such as young parents (often mothers) who had to learn to balance in new ways work and family responsibilities.^27^ While the pandemic has led many to return to live in the parental home, evidence so far did not support that changes in living arrangements at the start of the pandemic contributed to increased mental distress among young adults, suggesting that young adults may have appreciated to be with their parents in the context of the pandemic.^25 26^ Whereas the level of distress has been higher and access to health services has been further disrupted in deprived areas following the first lockdown, no studies that we know of have examined how socioeconomic background and area deprivation have influenced the mental health of young adults during the pandemic.^28 29^ One study found no ethnic inequalities in changes in psychological distress in women, but higher increases in South Asian men compared with white British men.^30^ Supporting this, some minority groups have been more likely to be working in shutdown sectors, in precarious employment, self-employed with less stable incomes and have fewer savings.^2 31^

### Objectives

Evidence on which young adult groups have been most at risk of poor mental health has been lacking. This study aims to (1) report changes in psychological distress among those aged 16–24 over the past decade and during the pandemic in England, using a survey repeated annually between 2009 and 2019 and six additional times in 2020; (2) examine the extent to which long-term trends and changes in 2020 varied across transition (economic activity and cohabitation with parents) and background (parental education, area deprivation, ethnicity, age and sex) characteristics; and (3) if changes in 2020 varied across background characteristics, examine if these could be attributable to changes in economic activity (ie, loss of job and work hours).

## Methods

### Data

We used data from the UK Household Longitudinal Study (UKHLS), a nationally representative household panel study of over 40 000 UK households that started in 2009.^32 33^ All those aged 16+ in contacted households were eligible for adult interviews. The fieldwork period for the main survey spans 24 months, with participants reinterviewed annually by online, face-to-face or telephone survey. In April 2020, a parallel COVID-19 survey was started with online surveys conducted with sample members aged 16+, repeated on a monthly basis from April to July and every two months afterwards.^34^ We used data from waves 1–10 of the main survey (from 2009–2010 to 2018–2019) and waves 1–6 of the COVID-19 survey (April–November 2020). The study sample comprised all those living in England, aged 16–24 at the interview date, with data on psychological distress, and a non-zero survey weight. Analyses were restricted to England as relative area deprivation measures (Index of Multiple Deprivation, IMD) are not directly comparable across UK countries. Sample sizes varied in the main waves from 4587 in wave 1 to 2333 in wave 10, and in the COVID-19 waves from 575 in April 2020 to 263 in November 2020 (online supplemental table 2).

10.1136/jech-2021-217266.supp1 Supplementary data

### Measures

*Psychological distress* was measured using the 12-item General Health Questionnaire (GHQ), a screening tool for non-psychotic and minor psychiatric disorders in the general population.^35^ The GHQ focuses on the inability to carry out normal function and the appearance of new and distressing phenomena (see items in online supplemental table 1). We used the GHQ score ranging from 0 (healthy) to 36 (fully distressed) based on the summation of the 12 items on their 4-point Likert scale (0–3). As a reference point, the SD of GHQ scores among those aged 16–24 varied between 6.2 and 6.8 across COVID-19 waves.

The characteristics used to examine distress over time included economic activity and cohabitation with parents as transition variables, and parental education, area deprivation, ethnic group, age and sex as background variables.

*Economic activity* was first collapsed into five categories: employed full time, employed part time, unemployed, full-time student and out of the labour force (eg, providing family care, not looking for work). In analyses only using the COVID-19 waves, *change in economic activity since before the pandemic* was then collapsed into four groups: (1) did not lose their job, (2) lost their job or work hours by 50% or more, (3) started a job, and (4) did not work before the pandemic and at the interview date. To assess economic activity before the pandemic, the questionnaires included retrospective questions on work in January–February 2020. We did not include furlough status in the ‘change in economic activity’ variable as too few participants reported this (from a high of 17% in the April wave down to 3%–6% in subsequent waves).

*Cohabitation with parents* was derived from the household grid to indicate if the respondent lived with at least one biological, adoptive or step-parent at the interview (yes/no). Students not living with their parents at the interview date were therefore not defined as cohabiting with parents. The COVID-19 questionnaires did not include retrospective questions on living arrangements before the pandemic, precluding us from investigating changes in living arrangements since before the outbreak.

*Parental education* was obtained from parents if respondents lived with them in at least one wave and from respondents themselves if they never lived with parents over the course of the study, and this was collapsed into two groups: at least one parent has a higher education degree and no degree. For *area deprivation*, we use information on the Lower Super Output Area (LSOA; an area of around 600 households) of the respondents and merged it with the 2010 English Index of Multiple Deprivation to derive area deprivation quartiles at the LSOA level.

Finally, *ethnic group* was collapsed into seven categories: (1) white UK, (2) white other and Irish, (3) mixed, (4) Indian, (5) Pakistani and Bangladeshi, (6) black Caribbean, African and other, and (7) all other ethnic groups.

We finally used data on *age* at the time of interview (16–18, 19–21, 22–24) and *sex* (male, female). Descriptive statistics and missing cases are detailed in online supplemental table 3.

### Statistical analyses

We first estimated mean GHQ scores across the 10 main survey waves (from 2009–2010 to 2018–2019) and in the six COVID-19 waves (April–November 2020), pooled to increase statistical power, and repeated this across social variables. We also tested differences in mean GHQ scores by variables in wave 1 (n=4587), wave 10 (n=2333) and the pooled COVID-19 sample (n=2382 observations from 697 participants).

We then modelled changes in psychological distress across these three time points. We estimated two sets of models comparing (1) data from waves 1 and 10 to identify trends across the past decade and (2) data from wave 10 and the pooled COVID-19 sample to identify changes during the pandemic. Using pooled linear models, we included a time dummy (0/1) to estimate the average change across time points treated as repeated cross-sectional waves, adjusting for the transition and background variables to account for differences in demographics between waves over time. Other studies have used a similar approach to examine changes in GHQ score in the UKHLS main and COVID-19 waves.^7 36 37^ Next, we tested interactions between time and variables and estimated the average marginal effect (AME) of time within variable categories to examine differences in the magnitude of change in GHQ scores across groups over time. For trends across the past decade, we only used waves 1 and 10 to derive meaningful estimates of changes over average wave-specific changes. As sensitivity analyses, we reran (1) the models for trends across the past decade examining the average wave-based change across the 10 main waves (online supplemental table 4) and (2) the models for changes during the pandemic using both waves 9 and 10 in the ‘before’ category (online supplemental table 5). Both supported the findings presented here.

Models were estimated in complete-case samples using Stata V.16.^38^ All estimates were produced using the weights provided by UKHLS to account for unequal selection probabilities and non-response. We accounted for the clustering and stratification of the sample design and the clustering of individuals to produce correct SEs.

If differences in GHQ scores varied across background variables during the pandemic (ie, between the wave 10 and pooled COVID-19 samples), we wanted to identify the potential contribution of transition characteristics through changes in economic activity. We therefore estimated a final set of models in the pooled COVID-19 sample (April–November 2020) only. We replaced in these models current activity with ‘changes in economic activity compared with before the pandemic’, and regressed GHQ scores in the pooled COVID-19 sample focusing on the background variable(s) showing increased differences in GHQ scores across categories during the pandemic. This was done in two models without and with the ‘changes in economic activity’ variable, controlling each time for other covariates. As those with higher levels of mental distress may have been affected differently by the pandemic compared with those with lower levels of mental distress, we also included the GHQ score measured at wave 10 as one of the covariates in these models. To integrate the repeated nature of observations in the pooled COVID-19 sample, we used in this final step random-intercept models in the participants who responded in all waves, using the November 2020 longitudinal weight. Since using this longitudinal weight reduced the pooled COVID-19 sample size by 48% (complete-case: from n=2049 to n=1069) compared with cross-sectional weights, we also reproduced this analysis using the same modelling approach as in the previous models (ie, pooled linear models with wave-specific cross-sectional weights) in online supplemental table 6.

## Results

Table 1 presents the mean GHQ scores in the three samples for 2009–2010, 2018–2019 and 2020 across groups (GHQ scores across the 10 main waves are presented in online supplemental figures). Psychological distress increased across time points, with mean GHQ scores increasing from 10.4 in 2009–2010 to 12.1 in 2018–2019 and 14.0 in 2020. In 2009–2010, psychological distress was significantly higher for those aged 19–21 and 22–24, women, those unemployed and out of the labour force, and those in the mixed ethnic group. In 2018–2019, sex and economic activity continued to be associated with psychological distress, but there were no more differences by age and new differences by ethnicity, with those in the white UK and white other groups reporting higher distress and those in the black group reporting lower distress. In 2020, (1) sex and economic activity continued to be associated with psychological distress; (2) differences by ethnicity changed, with those in the mixed ethnic group reporting again higher distress; and (3) there were new differences by area deprivation, with those in the most deprived area reporting higher distress.

**Table 1:** Psychological distress among young adults aged 16–24 living in England

Subgroups	Mean GHQ-12 score (range: 0–36) Full sample		
	2009–2010 (n*=*4587)	2018–2019 (n=2333)	April–November 2020 (n=2382)
All	10.4	12.1	14.0
Sex			
Male	**9.6**	**11.0**	**12.4**
Female	**11.1**	**13.0**	**15.2**
Age			
16–18	**9.5**	12.3	13.3
19–21	**10.5**	11.7	13.7
22–24	**11.0**	12.2	14.3
Economic activity			
FT employed	**10.1**	**11.2**	**13.0**
PT employed	**10.2**	**12.3**	**14.0**
Unemployed	**11.1**	**13.4**	**15.2**
FT education	**10.0**	**11.7**	**13.2**
Out of labour force	**12.5**	**15.8**	**16.1**
Living arrangements			
With parent(s)	10.0	12.0	14.0
Not with parent(s)	10.9	12.5	13.7
Parental education			
Degree	10.4	11.9	13.5
No degree	10.2	12.2	14.3
Area deprivation			
Most deprived	10.2	12.2	**15.4**
Second most deprived	10.5	12.0	**13.9**
Second least deprived	10.5	12.3	**13.7**
Least deprived	10.1	11.7	**13.3**
Ethnicity			
White UK	**10.4**	**12.3**	**14.0**
White other	**10.5**	**12.6**	**14.6**
Mixed	**11.7**	**11.1**	**16.0**
Indian	**9.9**	**11.3**	**14.0**
Pakistani and Bangladeshi	**10.2**	**10.6**	**13.3**
Black	**9.6**	**9.1**	**9.2**
Other	**10.4**	**11.0**	**13.9**

Understanding Society, 2009–2010, 2018–2019 and 2020.

GHQ scores that differed across categories at p<0.05 are in bold.

FT, full time; GHQ-12, 12-Item General Health Questionnaire; PT, part time.

Table 2 presents the results from the fully adjusted linear models testing the differences in mean GHQ scores between these time points. We found significant differences across three variables for changes in psychological distress between 2009–2010 and 2018–2019: (1) a larger increase in women compared with men (AME_W_=2.1 vs AME_M_=1.3); (2) a larger increase in those aged 16–18 compared with older young adults (AME_16–18_=2.6 vs AME_19–21_=1.2 and AME_22–24_=0.9); and (3) a larger increase in white UK, white other and Indian groups (AME_WUK_=2.0, AME_WOTH_=2.1, AME_IND_=1.5) compared with other ethnic groups (AMEs ranging from −1.0 to 0.4). We also found weak evidence (global p=0.103) of larger increases in distress among those in part-time employment (AME=2.2, p=0.049) and out of the labour force (AME=3.6, p=0.045) compared with those in full-time employment (AME=0.8).

**Table 2:** Testing changes in psychological distress over time among young adults aged 16–24 living in England, by different subgroups

Subgroups	Between 2009–2010 and 2018–2019 (n*=*5873)			Between 2018–2019 and April–November 2020 (n*=*4302)		
	AME of time on GHQ	95% CI	Interaction p value	AME of time on GHQ	95% CI	Interaction p value
All	**1.66**	**1.25 to 2.07**		**1.96**	**1.27 to 2.66**	
Sex			**0.024**			0.364
Male	**1.25**	**0.73 to 1.76**		**1.65**	**0.64 to 2.66**	
Female	**2.09**	**1.51 to 2.66**		**2.26**	**1.26 to 3.16**	
Age			**<0.001**			0.695
16–18	**2.64**	**2.04 to 3.23**		1.28	−0.71 to 3.26	
19–21	**1.15**	**0.53 to 1.78**	**0.001**	**2.02**	**1.10 to 2.94**	0.474
22–24	0.86	−0.01 to 1.72	**0.001**	**2.23**	**1.21 to 3.25**	0.396
Economic activity			0.103			0.899
FT employed	**0.84**	**0.11 to 1.58**		**1.89**	**0.93 to 2.85**	
PT employed	**2.16**	**1.05 to 3.27**	**0.049**	**1.88**	**0.71 to 3.04**	0.991
Unemployed	**2.15**	**0.68 to 3.62**	0.114	1.95	−0.57 to 4.47	0.962
FT education	**1.61**	**1.11 to 2.11**	0.083	**2.03**	**0.76 to 3.30**	0.851
Out of labour force	**3.59**	**0.99 to 6.20**	**0.045**	0.41	−2.50 to 3.31	0.343
Living arrangements			0.861			0.447
With parent(s)	**1.68**	**1.26 to 2.09**		**2.04**	**1.29 to 2.78**	
Not with parent(s)	**1.54**	**0.08 to 3.01**		1.41	−0.06 to 2.88	
Parental education			0.117			0.916
Degree	**1.87**	**1.36 to 2.39**		**1.93**	**1.04 to 2.81**	
No degree	**1.24**	**0.62 to 1.86**		**1.99**	**1.10 to 2.87**	
Area deprivation			0.642			**0.021**
Most deprived	**1.29**	**0.46 to 2.12**		**4.11**	**2.41 to 5.81**	
Second most deprived	**1.92**	**1.11 to 2.74**	0.525	**1.36**	**0.22 to 2.51**	0.454
Second least deprived	**1.53**	**0.77 to 2.29**	0.902	**1.80**	**0.44 to 3.15**	0.823
Least deprived	**1.86**	**1.16 to 2.55**	0.301	**1.21**	**0.30 to 2.11**	**0.002**
Ethnicity			**0.006**			0.394
White UK	**1.96**	**1.50 to 2.42**		**1.78**	**0.98 to 2.59**	
White other	2.08	−0.68 to 4.84	0.933	2.55	−1.61 to 6.71	0.722
Mixed	−0.24	−1.56 to 1.10	**0.002**	**4.43**	**2.04 to 6.81**	**0.037**
Indian	1.54	−0.38 to 3.46	0.673	2.28	−0.69 to 5.24	0.755
Pakistani and Bangladeshi	0.35	−1.03 to 1.74	**0.029**	**2.25**	**1.05 to 3.46**	0.514
Black	0.04	−1.68 to 1.77	**0.033**	0.87	−1.77 to 3.51	0.510
Other ethnic groups	−0.98	−2.75 to 0.79	**0.002**	**3.60**	**0.69 to 6.51**	0.234

Understanding Society, from 2009–2010 to 2018–2019 and from 2018–2019 to 2020.

Estimates come from fully adjusted linear models in pooled samples of observations using the wave-specific cross-sectional weights, clustering on individuals. Interactions were then tested for each variable in separate models. AMEs are based on complete-case models adjusted for all other predictors (age, sex, economic activity, living arrangements, parental education, area deprivation and ethnicity).

P values reported next to variable names refer to global tests of differences in AMEs across categories. P values reported next to variable categories refer to test of differences in AMEs with respect to the reference group.

Significant estimates at p<0.05 are in bold.

AME, average marginal effect; FT, full time; GHQ, General Health Questionnaire; PT, part time.

Differences were significant for one variable with regard to changes in psychological distress between 2018–2019 and 2020: area deprivation. A larger increase was found among those living in areas in the most deprived quartile (AME=4.1) compared with areas in the least deprived quartile (AME=1.2). We also found weak evidence of larger increases in distress among those from a mixed ethnic group (AME=4.4, interaction p=0.037) compared with those from white UK group (AME=1.8).

Table 3 presents the association of area deprivation with psychological distress in the pooled COVID-19 sample before and after adjustment for changes in economic activity compared with before the outbreak. Across COVID-19 waves, 35% of observations reported that they remained employed with similar work hours, 24% reported having lost their employment or 50% or more of their work hours, 7% had started a job, and 34% did not work both before the pandemic and at the interview date. In the baseline model adjusted for other social variables and GHQ score at wave 10, young adults living in an area in the highest deprivation quartile in 2020 had a 2.1 higher GHQ score (95% CI 0.9 to 3.3) compared with those in the lowest deprivation quartile. In the full model including changes in economic activity, those living in an area in the most deprived quartile had a 1.8 higher GHQ score (95% CI 0.5 to 3.0). In the full model, compared with those who remained employed with similar work hours, those who lost their job or 50% or more of their work hours had a 1.5 higher GHQ score (95% CI 1.0 to 2.0) and those who started a job reported a 2.7 lower GHQ score (95% CI –3.6 to −1.7). Contrasting estimates between the baseline and full models, including changes in economic activity since before the outbreak, attenuated the differences of those in the most deprived quartile by 17% (from *B*=2.10 to *B*=1.75) compared with those in areas in the least deprived quartile.

**Table 3:** Differences in psychological distress by area deprivation among young adults aged 16-24 living in England, considering economic changes since before the outbreak, UKHLS, April–November 2020

	Baseline model (n=1069) *B* (95% CI)	Model+job loss (n=1069) *B* (95% CI)
Area deprivation		
Most deprived	**2.10 (0.88 to 3.32)**	**1.75 (0.52 to 2.98)**
Second most deprived	0.78 (−0.23 to 1.78)	0.70 (−0.31 to 1.72)
Second least deprived	0.15 (−0.61 to 0.91)	−0.13 (−0.91 to 0.64)
Least deprived (reference)	–	–
Change since before outbreak		
Did not lose job or ≥50% hours (reference)	–	–
Lost job or ≥50% hours	–	**1.52 (1.01 to 2.02)**
Started job	–	**−2.65 (−3.58 to −1.72)**
Did not work at both points	–	0.37 (−0.39 to 1.30)

Estimates represent betas from random-intercept linear models using the UKHLS COVID-19 November 2020 longitudinal weight. The models controlled for wave, age, sex, parental education, living arrangements, ethnicity and GHQ score at wave 10.

Significant estimates at p<0.05 are in bold.

GHQ, General Health Questionnaire; UKHLS, UK Household Longitudinal Study.

## Discussion

This study highlights the worrisome trend of increasing psychological distress among young adults aged 16–24 years old in England over the past decade. The mechanisms underlying this long-standing trend are complex, but likely include the precarisation of the labour market (and its spillover effects on family transitions) that started in the 1990s, was exacerbated by the Great Recession in 2008–2009 and worsened over the first months of the COVID-19 pandemic.^12^ The findings support the presence of inequalities in mental health in this age group that have persisted over the past decade and increased during the pandemic. Between 2009–2010 and 2018–2019, psychological distress increased more in women, in those aged 16–18, and in white UK, white other and Indian groups. There was also evidence of increased distress in young adults employed part time and out of the labour force compared with those in full-time employment. However, we found no significant differences, or changes in differences over time, for the other indicators: that is, cohabitation with parents, parental education and area deprivation. This suggests that, despite the stagnating incomes and worsening conditions experienced in this age group over time, employment remains a key factor in shaping the mental health of young adults in recent years.^15^

Inequalities in mental health were exacerbated in new ways during the pandemic. Notably, increases in psychological distress have been 3.4 times larger in young people living in the most deprived areas compared with those in the least deprived areas. Studies that have associated COVID-19 cases and deaths with area deprivation highlighted occupational exposure, overcrowding, public transport use and underlying health conditions as mechanisms, which may also explain the unequal increases in psychological distress found here.^39^ Since lockdown measures prevented young adults from leaving their residential area, the conditions found in the most deprived areas may have had a stronger influence on those previously able to access less deprived areas in their everyday activities.^40^

Supporting the role of the economic consequences of the pandemic in mental health, we found that losing one’s job or work hours was related to increased psychological distress. In the UK, policies such as the Coronavirus Job Retention Scheme (ie, ‘furlough’) were rapidly implemented to protect wages. Unfortunately, preliminary studies suggest that these may have had a limited role in mitigating the effects of reduced hours on mental distress, at least in the short term.^41^ Changes in economic activity were also linked to the role of area deprivation in mental health in this group, attenuating about 17% of differences between those living in more and less deprived areas. The pandemic thus impacted on population health through mechanisms not formally addressed in this study (eg, fear of infection, social isolation, housing conditions) that may subside as the pandemic ends, and via the disruption of employment opportunities, which may have consequences for years to come. The lack of opportunities in more deprived areas may stem from the lack of highly skilled jobs, a weak fit between education and local employment conditions, and underfunded public resources diverted away from smaller towns in recent decades.^42^ Learning from the evidence on the impact of economic crises such as with the 2008 Great Recession, we anticipate the new pressures made on young adults to be associated with short-term increases in mental health problems as well as long-term ‘scarring effects’ over their life course.^6 43 44^

### Strengths and limitations

This study benefits from the strengths of the UKHLS to report representative trends in psychological distress among those aged 16–24 living in England over the past decade and during the pandemic in 2020, but is not without limitations. The COVID-19 waves had relatively low response rates and small young adult samples, precluding us from stratifying analyses by sex. The design of the main and COVID-19 surveys affected the composition of samples across waves (eg, respondents were more likely to be living with parents at wave 10 compared with wave 1 and less likely to be aged 16–18 in the COVID-19 waves), which may have biased the results despite statistical adjustment. Whereas data on many parental characteristics were available, parental education was the only measure with an acceptable level of missingness across waves. Including parental education removed more young adults living without parents in the complete-case analyses; however, findings were similar when this variable was removed from the models.

## Conclusion

Young people’s mental health has decreased considerably over the last decade and shows persistent inequalities by gender and economic activity. The COVID-19 pandemic has created new inequalities, with increased levels of distress found among young people living in more deprived areas in 2020. Supporting young people requires a holistic approach, which includes an appreciation of the diversity of their experiences by age, gender, social origin and ethnicity. Addressing this requires (1) a better understanding of the mechanisms leading to rising levels of distress in young people; (2) interventions reducing pressures on young people, such as promoting viable employment and housing opportunities, as well as investments in deprived areas; and (3) policy approaches integrating efforts directed at the individual, family and community levels to address the structures that shape young people’s opportunities for better health.

**What is already known on this subject**

- Studies have highlighted increases in mental health problems among young adults aged 16–24 in England both over the past decade and at the start of the COVID-19 pandemic in 2020 compared with older age groups.
- There has, however, been a paucity of evidence on the differences in these changes across social groups over time.

**What this study adds**

- The pandemic has accelerated pre-existing social inequalities by gender, economic activity and ethnicity, with higher levels of psychological distress found among young adults living in the most deprived areas in 2020 compared with prepandemic estimates.

## Data Availability

Data are available in a public, open access repository.
