# Supplementary material for: Time trend analysis of social inequalities in psychological distress among young adults before and during the pandemic: evidence from the UK Household Longitudinal Study COVID-19 waves
Source: J Epidemiol Community Health. 2021 Oct 29;76(5):421–7. doi: 10.1136/jech-2021-217266 (PMC8561821; doi:10.1136/jech-2021-217266)
Supplement: Supplementary data [file jech-2021-217266supp001.pdf]

SUPPLEMENTARY MATERIAL

Last updated August 25th, 2021

1. GHQ-12 item labels
2. Sample size per wave
3. Sample characteristics across waves 1, 10, and the pooled COVID sample
4. Re-analysis I – Trends in GHQ scores
5. Re-analysis II – Changes in GHQ scores before/during COVID
6. Re-analysis III – Differences in GHQ scores during the pandemic
7. Supplementary Figures of trends in GHQ score between UKHLS Waves 1 through 10

Gagné T, *et al. J Epidemiol Community Health* 2022; 76:421–427. doi: 10.1136/jech-2021-217266

SUPPLEMENTARY TABLE 1
GHQ-12 item labels

#	Full item
1	Have you recently been able to concentrate on whatever you're doing?
2	Have you recently lost much sleep over worry?
3	Have you recently felt that you were playing a useful part in things?
4	Have you recently felt capable of making decisions about things?
5	Have you recently felt constantly under strain?
6	Have you recently felt you couldn't overcome your difficulties?
7	Have you recently been able to enjoy your normal day-to-day activities?
8	Have you recently been able to face up to problems?
9	Have you recently been feeling unhappy or depressed?
10	Have you recently been losing confidence in yourself?
11	Have you recently been thinking of yourself as a worthless person?
12	Have you recently been feeling reasonably happy, all things considered?

GHQ items are introduced with the preamble: “The next questions are about how you have been feeling over the last few weeks”.

SUPPLEMENTARY TABLE 2
Sample size

Survey	Cycle	Date	Sample size with valid cross-sectional weight	Sample size with valid cros— sectional weight and valid GHQ score
			N	*N*
Main	Wave 1	2009-10	4,731	4,587
	Wave 2	2010-11	4,217	4,178
	Wave 3	2011-12	3,803	3,797
	Wave 4	2012-13	3,485	3,481
	Wave 5	2013-14	3,187	3,186
	Wave 6	2014-15	2,879	2,859
	Wave 7	2015-16	3,475	3,451
	Wave 8	2016-17	2,997	2,961
	Wave 9	2017-18	2,615	2,554
	Wave 10	2018-19	2,397	2,333
COVID	Wave 1	April 2020	743	575
	Wave 2	May 2020	483	457
	Wave 3	June 2020	419	402
	Wave 4	July 2020	397	378
	Wave 5	Sept. 2020	319	307
	Wave 6	November 2020	286	263

Universe: Participants aged 16-24 at current wave

SUPPLEMENTARY TABLE 3
Sample characteristics.

	W1		W10		Pooled COVID	
	2009-10		2018-19		2020	
**Variables**	W% Full *N* = 4,587	W% CC *N* = 3,811	W% Full *N* = 2,333	W% CC *N* = 2,062	W% Full *N* = 2,382	W% CC *N* = 2,240
**GHQ-12**						
Score out of 36 (Mean)	10.4	10.3	12.1	11.9	14.0	14.0
Missing (N)	0		0		0	
**Sex**						
M	49.3	49.1	50.8	51.1	55.1	54.3
F	50.7	50.9	49.2	48.9	44.9	45.7
Missing (N)	0		0		0	
**Age**						
16-18	33.0	37.4	34.8	37.5	8.2	8.0
19-21	32.8	31.8	34.5	35.4	42.6	42.8
22-24	34.1	30.8	30.7	27.1	49.2	49.2
Missing (N)	0		0		0	
**Economic activity**						
FT employed	25.3	25.2	23.3	21.7	24.5	24.7
PT employed	14.3	14.2	15.4	15.3	33.3	33.0
Unemployed	12.2	11.9	10.3	10.5	5.9	5.5
FT education	42.3	44.3	44.9	48.5	20.3	21.1
Other	5.6	4.5	3.8	4.0	15.2	15.6
Missing (N)	0		3		0	
**Living arrangements**						
With parent(s)	63.1	73.1	88.9	91.6	84.1	85.2
Not with parent(s)	36.9	26.9	11.1	8.4	15.9	14.8
Missing (N)	0		0		0	
**Parent education**						
Degree	27.7	27.7	37.3	37.5	38.0	37.6
No degree	72.3	72.3	62.7	62.5	62.0	62.4
Missing (N)	691		110		46	
**Area deprivation**						
Most deprived	22.2	23.8	26.0	26.7	31.9	32.7
2nd most deprived	25.2	25.6	26.7	27.2	26.2	26.3
2nd least deprived	27.3	26.3	24.7	24.2	21.0	20.8
Least deprived	25.2	24.3	22.6	21.9	21.0	20.2
Missing (N)	0		0		5	
**Ethnicity**						
White UK	78.9	80.5	79.5	80.2	74.9	75.5
White Other	3.5	2.8	2.7	2.6	2.3	2.1
Mixed	2.6	2.7	4.1	4.0	5.8	5.8
Indian	4.1	3.8	2.9	2.8	5.1	5.2
Pakistani & Bangladeshi	4.2	4.3	4.8	4.6	8.9	8.7
Black	3.6	3.4	3.6	3.4	2.1	1.9
Other	3.0	2.4	2.5	2.4	0.9	0.9
Missing (N)	70		83		50	

Universe: UKHLS participants ages 16-24 in England with no missing data on GHQ and a valid cross-sectional weight. CC = Complete-case samples for analyses in the main text's Table 2. Proportions are weighted using the wave-specific cross-sectional weight.

SUPPLEMENTARY TABLE 4

Testing changes in psychological distress over time among young adults aged 16-24 living in England, by different sub-groups. Understanding Society, 2009-10 to 2018-19.

	**Between 2009-10 and 2018-19** (N = 28,356)		
**Sub-groups**	AME of time on GHQ	95%CI	Interaction *p*
**Sex**			
M (ref)	**0.12**	**0.08; 0.17**	---
F	**0.23**	**0.18; 0.28**	**< 0.001**
**Age**			
16-18 (ref)	**0.25**	**0.20; 0.30**	---
19-21	**0.16**	**0.10; 0.21**	**0.003**
22-24	**0.10**	**0.04; 0.17**	**< 0.001**
**Economic activity**			
FT employed (ref)	**0.11**	**0.06; 0.17**	---
PT employed	**0.19**	**0.11; 0.27**	0.094
Unemployed	**0.24**	**0.11; 0.37**	0.074
FT education	**0.18**	**0.13; 0.22**	0.088
Out of labor force	**0.34**	**0.12; 0.55**	**0.046**
**Living arrangements**			
With parent(s)	**0.18**	**0.07; 0.25**	0.705
Not with parent(s) (ref)	**0.16**	**0.14; 0.22**	---
**Parent education**			
Degree	**0.17**	**0.11; 0.22**	0.690
No degree (ref)	**0.18**	**0.14; 0.22**	---
**Area deprivation**			
Most deprived	**0.14**	**0.06; 0.21**	0.449
2nd most deprived	**0.22**	**0.15; 0.29**	0.376
2nd least deprived	**0.17**	**0.11; 0.24**	0.977
Least deprived (ref)	**0.17**	**0.12; 0.23**	---
**Ethnicity**			
White UK (ref)	**0.21**	**0.17; 0.25**	---
White Other	0.09	-0.10; 0.28	0.225
Mixed	0.11	-0.07; 0.28	0.253
Indian	0.08	-0.07; 0.22	0.084
Pakistani & Bangladeshi	-0.04	-0.17; 0.09	**< 0.001**
Black	0.05	-0.17; 0.08	**< 0.001**
Other ethnic groups	0.05	-0.25; 0.35	0.311

Estimates are from linear models regressing GHQ on time-varying characteristics in the ten UKHLS main waves from Wave 1 (2009-10) to Wave 10 (2018-19) and a time variable. The estimates are from pooled OLS clustering on individuals using cross-sectional weights. Estimates represent the average increase in GHQ between any two waves. Interactions were included in separate models, one at a time.
AME = Average marginal effect. CI = Confidence interval. We bolded significant AMEs at $p < .05$.
Interactions *p*-values reported in the row with the variable names refer to test of differences in AMEs across categories, and those reported next to variable categories refer to test of differences in AMEs with respect to the reference group.
AMEs are based on complete-case models adjusted for all other predictors.

SUPPLEMENTARY TABLE 5

Testing changes in psychological distress over time among young adults aged 16-24 living in England, by different sub-groups. Understanding Society, 2017-19 to 2020.

	Between Waves 9-10 and Apr.-Nov. 2020 (*N* = 6,534)		
**Sub-groups**	AME of time on GHQ	95%CI	Interaction *p*
**Sex**			
M (ref)	**1.52**	**0.58; 2.45**	---
F	**2.12**	**1.26; 2.98**	0.355
**Age**			
16-18 (ref)	1.17	-0.79; 3.13	---
19-21	**2.00**	**1.11; 2.88**	0.412
22-24	**1.86**	**0.93; 2.80**	0.529
**Economic activity**			
FT employed (ref)	**1.81**	**0.94; 2.68**	---
PT employed	**1.90**	**0.89; 2.90**	0.885
Unemployed	1.99	-0.50; 4.49	0.887
FT education	**1.95**	**0.75; 3.16**	0.837
Out of labor force	0.95	-1.77; 3.67	0.558
**Living arrangements**			
With parent(s)	**1.94**	**1.25; 2.63**	0.200
Not with parent(s) (ref)	1.05	-0.18; 2.27	---
**Parent education**			
Degree	**1.56**	**0.73; 2.40**	0.446
No degree (ref)	**1.99**	**1.77; 2.81**	---
**Area deprivation**			
Most deprived	**4.08**	**2.44; 5.72**	**0.003**
2nd most deprived	1.02	-0.02; 2.07	0.628
2nd least deprived	**1.53**	**0.24; 2.82**	0.803
Least deprived (ref)	**1.34**	**0.51; 2.18**	---
**Ethnicity**			
White UK (ref)	**1.57**	**0.82; 2.31**	---
White Other	3.36	-0.52; 7.24	0.373
Mixed	**4.00**	**1.32; 6.60**	0.086
Indian	**2.92**	**0.16; 5.69**	0.361
Pakistani & Bangladeshi	**2.59**	**1.41; 3.77**	0.147
Black	0.63	-1.71; 2.96	0.445
Other ethnic groups	**3.07**	**0.10; 6.04**	0.337

Estimates are from linear models regressing GHQ on time-varying characteristics in UKHLS Wave 9 (2017-18) and Wave 10 (2018-19), and UKHLS COVID waves 1-6 (April-November 2020), and a time (0/1) variable. The estimates are from pooled OLS clustering on individuals using cross-sectional weights. Estimates represent the average increase in GHQ between 2017-19 and 2020. Interactions were included in separate models, one at a time.
AME = Average marginal effect. CI = Confidence interval. We bolded significant AMEs at $p < .05$.
Interactions p-values reported next to variable categories refer to test of differences in AMEs with respect to the reference group. AMEs are based on complete-case models adjusted for all other predictors.

SUPPLEMENTARY TABLE 6
Differences in psychological distress by area deprivation among young adults aged 16-24 living in England, considering economic changes since before the outbreak. UKHLS, Apr-Nov 2020.

	**Baseline model** (*N* = 2,049)		**Model + Job loss** (*N* = 2,049)	
	B	95%CI	*B*	95%CI
**Area deprivation**				
Most deprived	1.72	-0.38; 5.17	1.46	-0.66; 3.59
2nd most deprived	0.52	-1.21; 1.38	0.31	-0.89; 1.52
2nd least deprived	0.31	-1.54; 0.94	0.02	-1.27; 1.30
Least deprived (ref.)	---	---	---	---
**Change since before outbreak**				
Did not lose job or ≥ 50% hours (ref.)	---	---	---	---
Lost job or ≥ 50% hours	---	---	**1.32**	**0.05; 2.58**
Started job	---	---	-1.64	-3.67; 0.38
Did not work at both points	---	---	0.26	-0.92; 1.44

Estimates represent betas from pooled OLS models clustering on individuals using the wave-specific UKHLS COVID cross-sectional weights. Models controlled for wave, age, sex, ethnic group, parental education, living arrangements with parents, and GHQ score at Wave 10. We bolded significant estimates at $p < .05$. CI = Confidence interval.

Gagné T, *et al. J Epidemiol Community Health* 2022; 76:421–427. doi: 10.1136/jech-2021-217266

SUPPLEMENTARY FIGURES

GHQ-12 mean scores (range 0-36) among young adults aged 16-24 living in England. UKHLS, 2009-10 to 2018-2019.

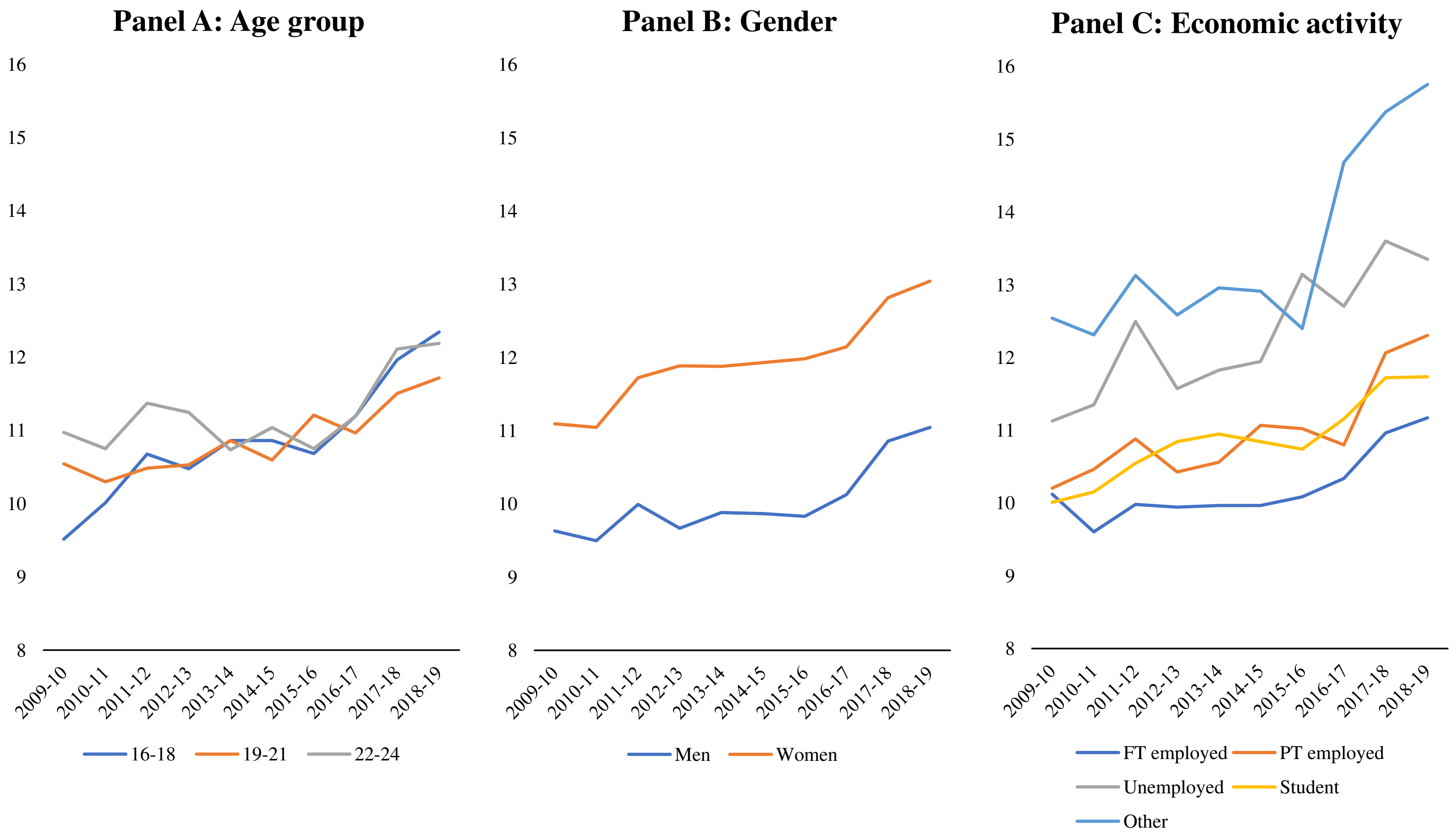

SUPPLEMENTARY FIGURES

GHQ-12 mean scores (range 0-36) among young adults aged 16-24 living in England. UKHLS, 2009-10 to 2018-2019.

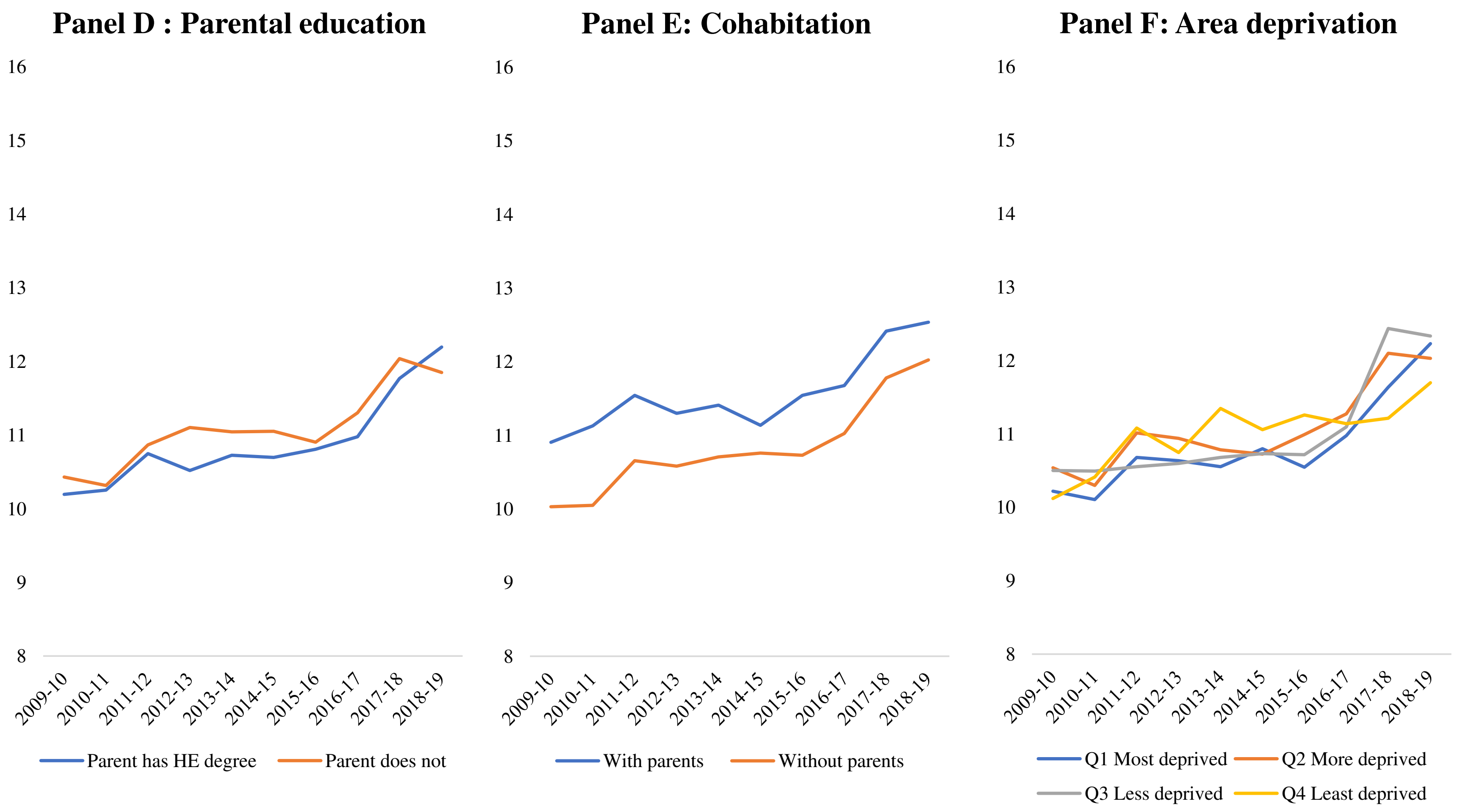

SUPPLEMENTARY FIGURES
GHQ-12 mean scores (range 0-36) among young adults aged 16-24 living in England. UKHLS, 2009-10 to 2018-2019.

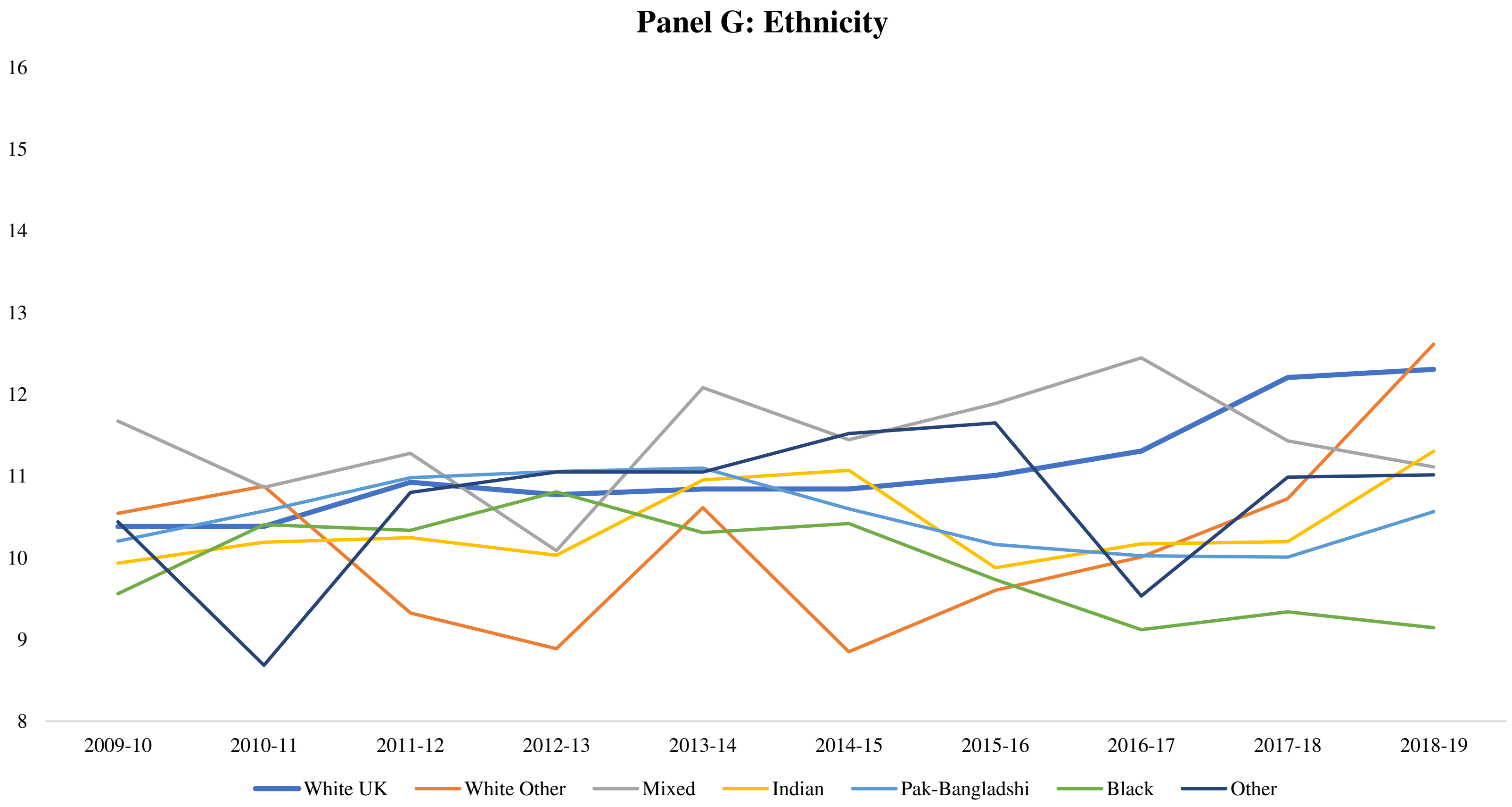

Gagné T, *et al. J Epidemiol Community Health* 2022; 76:421–427. doi: 10.1136/jech-2021-217266
